# Choreographing atomic motions of macromolecular machines in action: Towards dynamics‐based drug discovery

**DOI:** 10.1002/ctm2.977

**Published:** 2022-08-02

**Authors:** Shitao Zou, Youdong Mao

**Affiliations:** ^1^ State Key Laboratory for Artificial Microstructures and Mesoscopic Physics Institute of Condensed Matter and Material Physics, School of Physics Peking University Beijing China; ^2^ Peking‐Tsinghua Joint Center for Life Sciences Peking University Beijing China; ^3^ Center for Quantitative Biology, Academy for Advanced Interdisciplinary Studies Peking University Beijing China; ^4^ National Biomedical Imaging Center Peking University Beijing China

**Keywords:** cryo‐EM, deubiquitylating enzyme, drug discovery, nonequilibrium dynamics, proteasome, ubiquitin

## 1 INTRODUCTION

**FIGURE 1:**
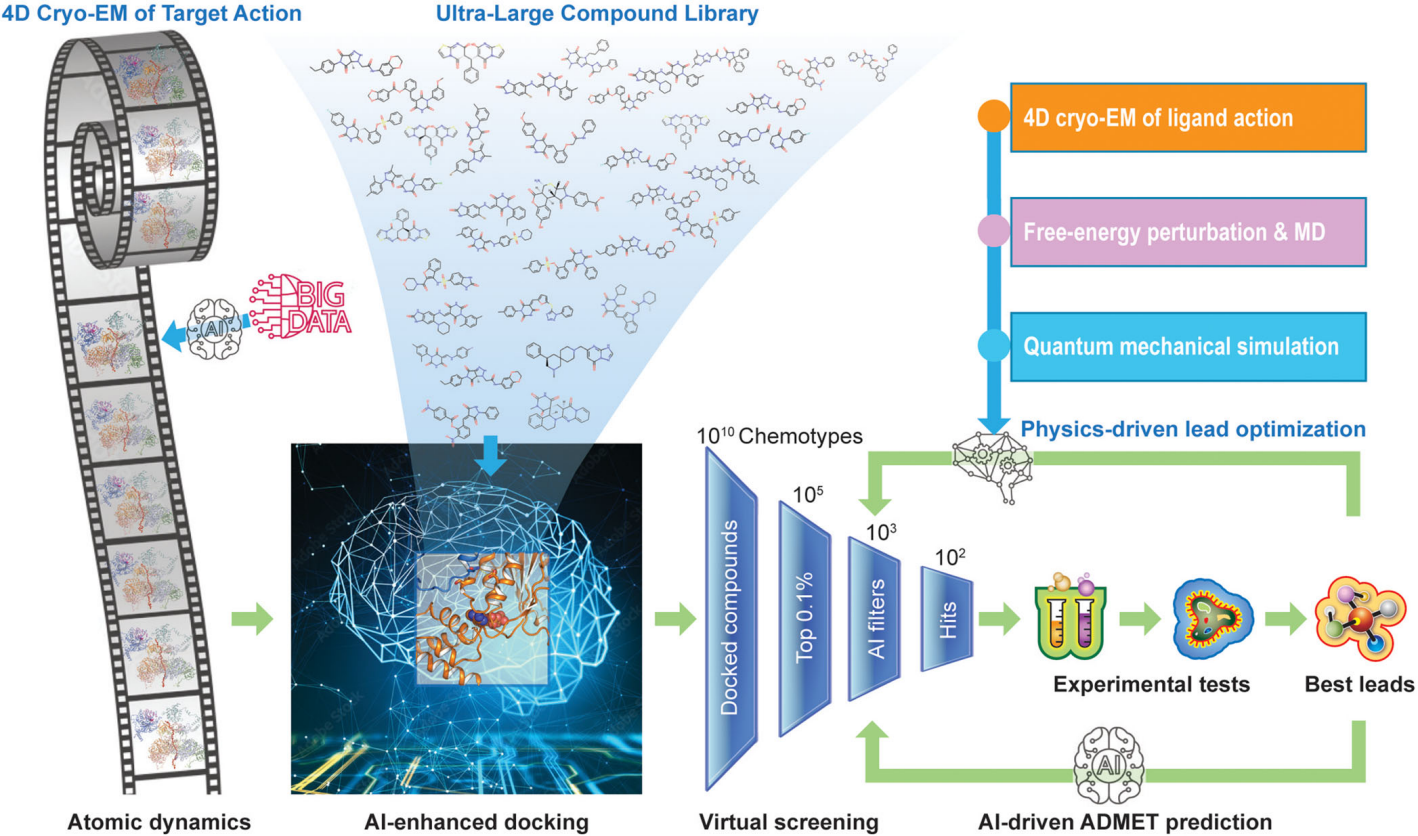
Application of time‐resolved or four‐dimensional cryogenic electron microscopy (4D cryo‐EM) to enable a dynamics‐based drug discovery process beyond traditional structure‐based drug discovery (SBDD). The atomic dynamics of therapeutic targets are obtained by large experimental datasets of 4D cryo‐EM and artificial intelligence (AI)‐enhanced data processing. Non‐equilibrium 3D conformations representing target dynamics in functional states provide realistic structural templates for molecular docking of ligands from ultra‐large compound libraries, which is expected to produce higher accuracy in ligand recognition and discovery of hits and leads. The virtual screening consists of an initial step of fast scoring of the ligand‐protein binding energy, followed by steps of AI‐driven filtering and ranking that narrow down to a few hundreds of hits. These hit compounds are then synthesized and tested in cell‐based and in vitro assays to identify the best lead compounds. The leads are further optimized by physics‐driven structural optimization that once again can benefit from the use of 4D cryo‐EM to verify the ligand action, in combination with complementary computational methods, such as free‐energy perturbation and quantum mechanical simulation

High‐resolution structures of macromolecules in functional states are the starting point for structure‐based drug discovery (SBDD). For decades, solving the atomic structure of a large macromolecular complex in a thermostable, equilibrated conformation was the major goal of understanding the structure‐function relationship. Researchers often optimistically assumed that all copies of the complex assume identical conformation and that such a conformation informs its physiological function in a native state. In the reality of a living cell, both assumptions are not well held in that macromolecules may suffer from a stochastic sampling of multiple conformations. Once encountering its substrates, ligands or activators, macromolecular complexes may enter nonequilibrium conformational transitions to execute the function. Thus, nonequilibrium atomic structures in functional states are considered superior to equilibrium structures in non‐functional states for mechanistic understanding and are expected to enable dynamics‐based drug discovery beyond SBDD (Figure 1).

## 2 CHALLENGE OF SOLVING NONEQUILIBRIUM STRUCTURES

Visualizing nonequilibrium dynamics of multi‐subunit holoenzymes in functional action at the atomic level has been largely inaccessible in the past. In such a process, many unstable or metastable intermediate conformations may be transiently formed. Atomic motions in the nonequilibrium intermediates are critical to understanding the inner working of biochemical processes and information on how a ligand or compound may bind to the active sites for altering its function. Unfortunately, their low abundance and extreme conformational heterogeneity make it very difficult or infeasible to visualize. This is particularly the case for a macromolecular complex with megadalton molecular weight.

Time‐resolved cryogenic electron microscopy (cryo‐EM) offers possibilities to break this bottleneck.^1^ However, several prerequisites must be met to make it feasible. First, a proper in vitro reconstruction of biochemical reaction must be worked out to sufficiently reproduce its physiological function in cells. This could be a daunting task for many cellular biochemical processes that are insufficiently understood. With only one missing reactant or activator, the biochemical reactions may never take place in vitro. Second, cryo‐EM reconstruction exercises image averaging from a large number of macromolecular copies. Image classification according to different conformations, often referred to as 3D classification, is plagued by the poor signal‐to‐noise ratio of raw cryo‐EM images, which mostly falls in the range of 0.005–0.05 or poorer. This severely limits how many different 3D conformations can be reliably sorted out and reconstructed to high resolution. Although structural biologists herald the so‐called “resolution revolution” in the cryo‐EM determination of equilibrium structures, solving dynamic motions of macromolecules by time‐resolved cryo‐EM has been previously limited to two to three coexistent conformations at moderate resolution.

## 3 FOUR‐DIMENSIONAL CRYO‐EM VISUALIZES ATOMIC MOTIONS OF MOLECULAR MACHINE

The ubiquitin‐proteasome system (UPS) is a primary proteolytic mechanism in eukaryotes, which mediates ATP‐dependent degradation of ubiquitin‐tagged proteins and regulates myriad cellular processes.^2^ Dysfunction of the proteasome is implicated in many human diseases, including cancer, inflammatory and neurodegenerative diseases.^2^ Targeted protein degradation by the proteasome is a highly dynamic, nonequilibrium process.^3^ Regulation of proteasome function represents a clinically proven strategy for cancer therapy, with three FDA‐approved proteasome inhibitors, including bortezomib, carfilzomib and ixazomib. A primary regulatory checkpoint in the UPS is the removal of ubiquitin tags by the deubiquitylating enzyme (DUB). Ubiquitin‐specific protease 14 (USP14) is the primary DUB reversibly associated with the proteasome and plays a pivotal role in the UPS.^2^ Proteasome regulation by USP14, which adds another layer of complexity to the intricacy of proteasome dynamics,^3^, ^4^ has defied mechanistic understanding by existent technology including traditional cryo‐EM.

**FIGURE 2:**
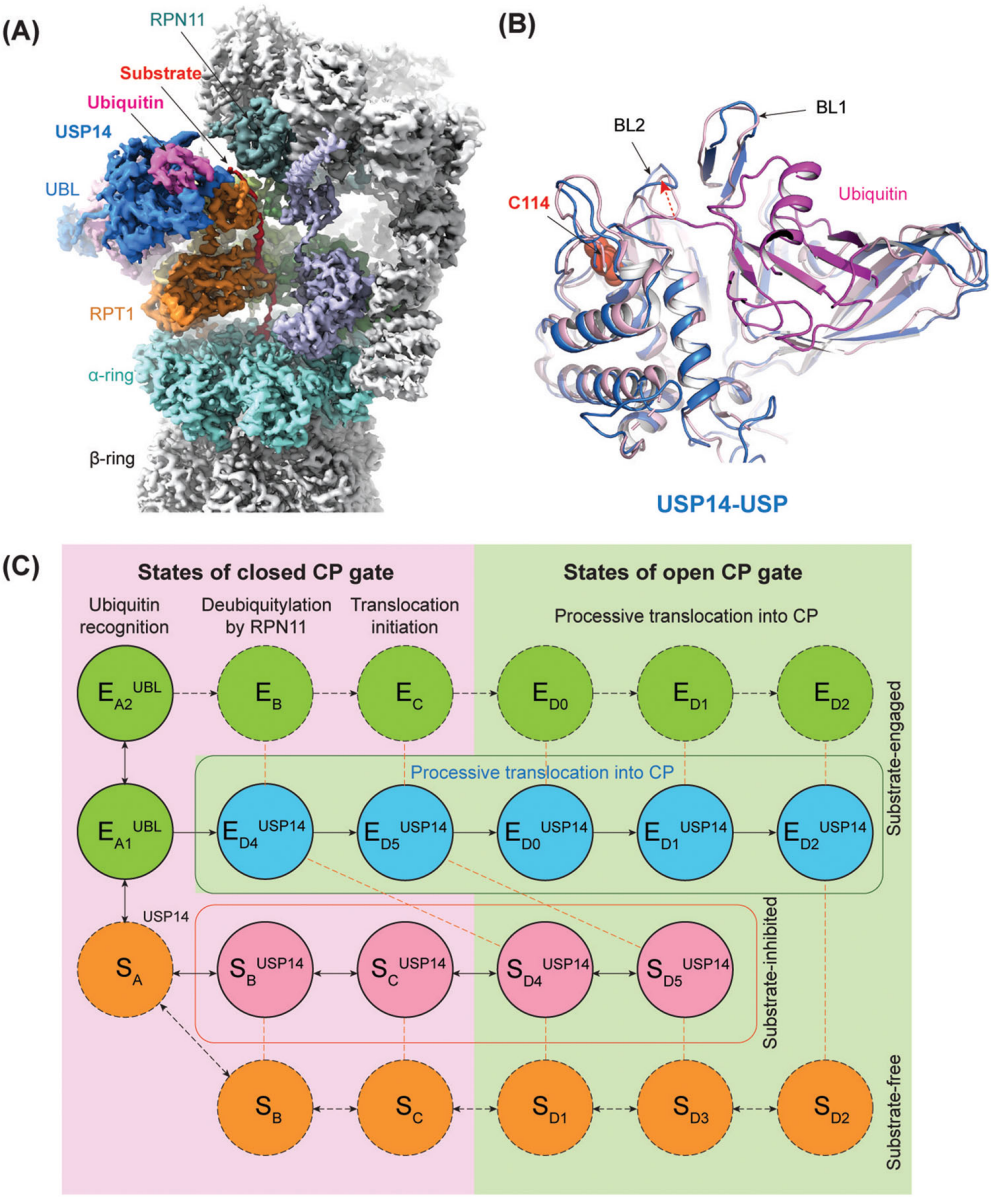
Four‐dimensional cryogenic electron microscopy (4D cryo‐EM) choreographs human ubiquitin‐specific protease 14 (USP14)–regulated proteasome in the nonequilibrium process of substrate degradation. (A) Cryo‐EM density map of the human USP14–proteasome complex in one of 13 conformational states captured in the act of substrate degradation. (B) Structural alignment of USP14 catalytic domain in the proteasome‐bound state (marine, PDB: 7W3H) with the crystal structure of the free, auto‐inhibited state (light pink, PDB: 2AYN), showing the marked movement of BL1 and BL2 loops upon USP14 activation by the proteasome. (C) An integrated schematic diagram illustrates how USP14 creates parallel pathways of proteasome state transitions and three regulatory checkpoints on the proteasome. At the step of initial ubiquitin recognition, USP14 allosterically competes against RPN11 in recruiting ubiquitylated substrate, creating the first checkpoint. During the initiation of substrate translocation by the AAA‐ATPase motor, the USP14 association drives the proteasome to choose between two alternative pathways, presenting the second checkpoint. The third checkpoint occurs at the recycling of ubiquitin chains, where USP14 delays ubiquitin release from the proteasome, thus non‐catalytically slowing down protein degradation. CP, core particle. *Source*: Figure adapted from reference 1

Recent advances in the development of a novel deep learning framework for high‐accuracy 3D classification^5^ overcame the major barrier in visualizing nonequilibrium dynamics of megadalton holoenzymes in action, allowing tens of conformers to be accurately classified and simultaneously resolved.^6^ This approach allowed us to advance time‐resolved or 4D cryo‐EM in solving 13 nonequilibrium conformers of the USP14‐regulated proteasome in the time‐resolved course of ubiquitylated protein degradation at 3–3.6 Å resolution (Figure 2).^1^ This work elucidated for the first time the molecular mechanism of human USP14 activation by the proteasome and how this is mutually translated into proteasome regulation at the atomic level.

## 4 CONTROL OF THE PROTEASOME REVEALS THERAPEUTIC TARGETS

Abnormal expression of USP14 is related to tumorigenesis and the progression of various cancers by regulating several signalling pathways. For example, USP14 activates the Wnt/β‐catenin pathway via stabilizing β‐catenin protein.^7^ Deubiquitylation of NLRC5 or RIG‐I by USP14 inhibits NF‐κB activation, while USP14 promotes the NF‐κB signalling activity via deubiquitylating IκBα for proteasomal degradation in lung epithelial cells.^8^ Interestingly, activation of Akt that could promote USP14 activity through phosphorylating USP14 at Ser432 was inhibited by b‐AP15, a selective USP14 inhibitor.^9^ Therefore, USP14 is considered a prognostic marker and therapeutic target for multiple cancers. A USP14‐selective inhibitor VLX1570 has shown remarkable activities in suppressing the growth of multiple myeloma and had been investigated in clinical trials (phase 1/2).^10^ Unfortunately, the trials failed due to the dose‐limiting toxicity of VLX1570 (ClinicalTrials.gov, Identifier: NCT02372240).

USP14 has also been demonstrated to be associated with neurodegenerative disorders, because it can modulate the clearance of protein aggregates, such as tau and TDP‐43 in neurons, through regulating proteasome activity or mediating the coordination between the UPS and autophagy. Thus, it is highly desirable that small‐molecule USP14 inhibitors could be developed into therapeutic drugs alleviating neurodegenerative diseases. Markedly, time‐resolved cryo‐EM in conjunction with functional studies revealed that USP14 regulates proteasome function by creating three regulatory checkpoints^1^ (Figure 2C). The novel insights inform about mechanistic strategies for developing highly selective inhibitors targeting USP14. Although computational simulation of molecular dynamics has been utilized to improve ligand docking in SBDD, the experimental dynamics of the USP14‐controlled proteasome in functional action^1^ provide realistic atomic target templates to enable dynamics‐based drug design with potentially improved accuracy (Figure 1).

## 5 OUTLOOK

The demonstration of solving atomic dynamics of nonequilibrium intermediates of the human USP14‐controlled proteasome,^1^ which is truly no less complex than ribosome and together governs the entire proteome homeostasis,^2^ will likely inspire more experimental studies to follow. Mechanisms of many other proteasome‐regulating proteins in the UPS, including numerous E3 ligases, may be determined in years to come, allowing more therapeutic targets to be revealed. Importantly, all the technical advances and improvements will eventually lead us to understand the nonequilibrium dynamic processes of everything happening in cells. Ultimately, a virtual cell could be built at the atomic level and all cellular biochemical reactions can be accurately reproduced in silico, given the atomic‐level knowledge we learned from time‐resolved atomic reconstructions of all cellular dynamics and macromolecular interactions. In the future, one could expect that all therapeutic drug candidates can be tested in a virtual cell in silico, the ultimate degree of virtual screening in drug discovery that might replace experimental cell‐based screening completely. While it is hard to predict how long it will take to achieve this feat, it is certain that the journey to an augmented future has already begun.
